# Physical activity patterns in people with haemophilia with and without arthropathy: a cross-sectional study using wearable sensors

**DOI:** 10.1186/s12959-026-00869-2

**Published:** 2026-05-07

**Authors:** Yuya Mawarikado, Midori Shima, Naoki Matsumoto, Asuka Sakata, Ryohei Kawasaki, Kohei Tatsumi, Tetsuhiro Soeda, Suguru Harada, Naoto Seriu, Yusuke Inagaki, Akira Kido, Keiji Nogami

**Affiliations:** 1 Medicinal Biology of Thrombosis and Haemostasis, Nara Medical University, 840 Shijo-Cho, Kashihara, Nara 634-8521 Japan; 2 Department of Rehabilitation Medicine, Nara Medical University, 840 Shijo- Cho, Kashihara, Nara 634-8521 Japan; 3 Medical Affairs Division, Product Research Department, Chugai Pharmaceutical Co., Ltd., 216 Totsukacho, Totsuka-ku, Yokohama City, Kanagawa 244-8602 Japan; 4 Department of Blood Transfusion Medicine, Nara Medical University, 840 Shijo-Cho, Kashihara, Nara 634-8521 Japan; 5 Advanced Medical Science of Thrombosis and Haemostasis, Nara Medical University, 840 Shijo-Cho, Kashihara, Nara 634-8521 Japan; 6 Department of Paediatrics, Nara Medical University, 840 Shijo-Cho, Kashihara, Nara 634-8521 Japan

**Keywords:** Haemophilia A, Haemophilia B, Joint diseases, Physical activity

## Abstract

**Background:**

In haemophilia, recurrent joint bleeding often results in joint destruction, which in turn leads to reduced physical activity (PA) and decreased quality of life (QOL). While maintaining high PA levels is important in the care of people with haemophilia (PwH), it remains unclear whether PwH without joint disease can maintain adequate activity levels.

**Aim:**

This descriptive cross-sectional study sought to compare moderate-to-vigorous physical activity (MVPA) levels among 19 PwH with haemophilic arthropathy (HA), 12 PwH without HA, and 15 non-PwH.

**Methods:**

A total of 46 males wore a triaxial accelerometer (wGT3X-BT, ActiGraph) during daily activities for seven consecutive days. A bout of MVPA was defined as at least 10 consecutive minutes of moderate-intensity or higher activity. MVPA was expressed as the percentage of the total wear time spent in MVPA, and the total number of MVPA bouts was also recorded.

**Results:**

Both PwH groups demonstrated significantly lower MVPA percentages and fewer MVPA bouts than the controls (*p* < 0.01). No MVPA bouts were recorded among 52.6% of PwH with HA versus 25% of PwH without HA. No significant differences were observed in sedentary or light activity between the PwH groups.

**Conclusions:**

PA levels were significantly reduced in PwH, regardless of joint status, suggesting that factors beyond joint damage, such as fear of bleeding or behavioural avoidance, may play a role. These findings highlight the need for individualized, evidence-based interventions to safely promote physical activity and improve long-term health outcomes in PwH.

## Background

Haemophilia is a disorder that is linked to the X chromosome and causes qualitative or quantitative abnormalities in coagulation factor VIII or IX [1]. Abnormalities in coagulation factors can cause unprovoked or provoked bleeding, with joints being the most common bleeding sites [2]. Repeated intra-articular bleeding causes structural abnormalities in the joint, leading to functional impairment and pain. This joint abnormality is called haemophilic arthropathy (HA) and is one of the major complications observed among people with haemophilia (PwH). HA is responsible for chronic pain experienced by 32–50% of PwH, thereby reducing physical activity (PA) and functionality [1, 3–5].

In recent years, significant advancements have been made in haemophilia treatment, including improvements in treatment methods such as prophylactic therapy and the development of new therapeutic agents, including nonfactor agents. Therefore, the incidence of HA has decreased significantly [6], with a particularly marked trend observed among individuals receiving new treatments from an early age. Additionally, the reduction in bleeding episodes accompanied by painful symptoms has led to a decrease in the tendency to avoid participation in sports and other physically demanding activities [7]. It is increasingly recognized that PA offers numerous clinical benefits for PwH. While a previous Cochrane review highlighted the potential for exercise interventions to reduce pain and improve joint range of motion, the evidence level was considered low due to the limited number of high-quality studies [8]. However, more recent studies have further supported the positive impact of structured physical activity on muscle strength, functional capacity, and quality of life in PwH [9]. Activities with a high risk of serious injury should be avoided; however, the benefits of maintaining high levels of PA are considered to outweigh the risks of bleeding associated with exercise [1], and achieving PA levels recommended by the World Health Organization [10] or World Federation of Hemophilia [1] is important for maintaining overall and musculoskeletal health. While healthy individuals serve as a benchmark for comparison, individualized PA strategies should focus on safely attaining these established health-oriented targets rather than simply matching the activity levels of a non-haemophilic population.

Although previous studies have often reported reduced PA levels among PwH, particularly those with existing arthropathy, findings in the literature are divergent. Based on a review of available evidence, many PwH—especially those on long-term prophylaxis—are reported to be as active as their healthy peers [11]. These discrepancies suggest that PA levels are not uniformly diminished but are significantly influenced by factors such as age, treatment regimens, and the severity of the bleeding phenotype. While there are reports suggesting an increasing trend in sports participation among young PwH without HA [12], our previous study reported that even in PwH without HA, walking speed was slower and gait was more impaired than in age-matched healthy controls [13]. While these gait deviations are likely multifactorial—potentially influenced by subtle joint changes or muscle weakness—they may also reflect reduced PA levels. These results are inconsistent with the assumption that PwH without HA have PA levels equivalent to those of healthy controls. In this study, we hypothesized that PwH with HA would have lower PA levels. Therefore, the aim of this study was to compare PA levels between PwH with and without HA, and to further compare these levels with those of healthy participants. This dual approach allows us to determine the impact of arthropathy on PA within the PwH population while also situating their activity levels relative to the healthy general population. These findings are expected to increase knowledge regarding PA levels among PwH and promote the development of appropriate PA strategies tailored to the modern PwH population, who experience less frequent bleeding due to advancements in treatment.

## Methods

### Study design and participants

This study adopted a descriptive cross-sectional design to quantitatively compare PA levels across three groups: the PwH with HA group, the PwH without HA group, and the non-haemophilic (non-PwH) group.

Male participants were recruited from Nara Medical University Hospital in Japan. The healthy participants (non-PwH controls) were recruited from among the employees of Chugai Pharmaceutical Co., Ltd. in Japan. To ensure the objectivity of the data, the recruitment and data collection processes were conducted in a blinded manner, such that the researchers (including those who are co-authors) and the participants remained anonymous to each other during the study period.

The inclusion criterion for PwH was the ability to walk independently without the use of walking aids (e.g., canes or braces). The exclusion criteria were as follows: the presence of orthopaedic disorders other than HA, or any respiratory or neuromuscular conditions affecting mobility. Similarly, the exclusion criteria for non-PwH individuals were the presence of orthopaedic, respiratory, or other mobility-impairing disorders. For PwH, those who had undergone lower-extremity joint surgery within the past year were also excluded to eliminate the influence of post-surgical recovery. Additionally, participants who engaged in high-activity occupations, such as professional or competitive athletes, or firefighters, were excluded from this study.

The severity of HA in each joint was assessed radiologically using the Arnold–Hilgartner staging (AHS) system [14]. An orthopaedic surgeon specializing in HA diagnosed the presence or absence of HA in the knee or ankle joints. Stages 4–5 indicated HA, and stages 0–3 indicated the absence of HA. Participants were categorized into the PwH with HA group if at least one of the four lower-extremity joints (bilateral knee and ankle joints) had an Arnold-Hilgartner score of 4 or higher. PwH who did not meet this criterion for any of the four joints were assigned to the PwH without HA group.

All participants were given a clear and comprehensive explanation of the study in accordance with the Declaration of Helsinki. The participants subsequently signed informed consent forms. Ethical approval for the study was obtained from both Nara Medical University (ID: 1979) and Chugai Ethics Committees (ID: E20019).

### Background characteristics

The following data were collected from the patients’ electronic medical records: age, height, weight, body mass index (BMI), severity of haemophilia on the basis of their factor levels in plasma (severe: ≤1%; moderate: 1%−5%; and mild: >5% to ≤ 40%), and information on their treatment regimen (formulation name of coagulation factor concentrates and infusion interval of the concentrates).

### Measurement of physical activity

**Fig. 1:**
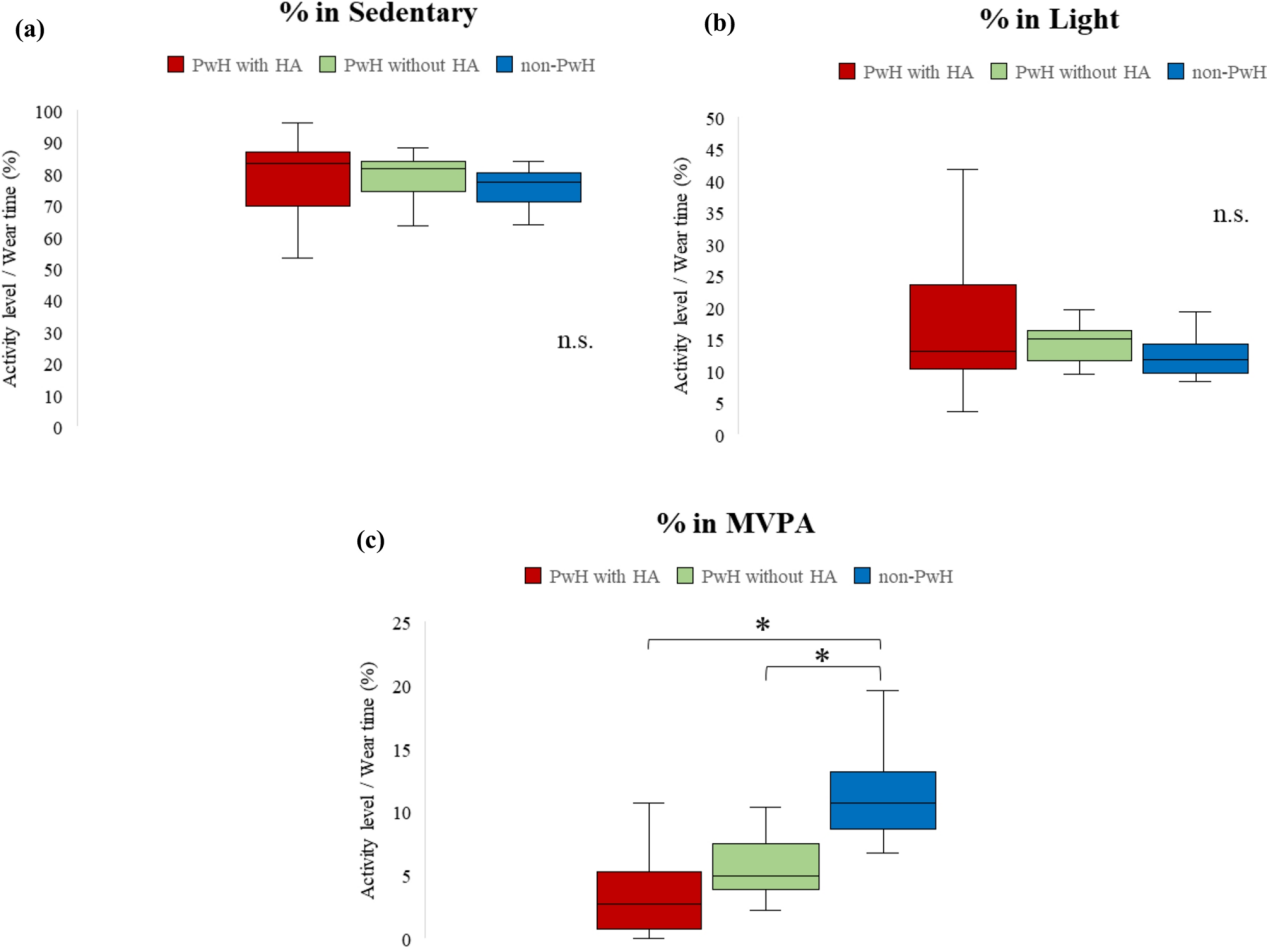
Activity levels among the three groups. (**a**) Percentage of sedentary time; (**b**) Percentage of light physical activity; (**c**) Percentage of moderate to vigorous physical activity (MVPA). Groups include PwH with HA (*n* = 19), PwH without HA (*n* = 12), and non-PwH (*n* = 15). The data are shown as box-and-whisker plots representing the interquartile range (IQR), median (centre line), and minimum/maximum values, excluding outliers. MVPA was significantly lower in both the PwH with HA and the PwH without HA groups than in the non-PwH controls. No significant differences were found in sedentary or light activity among the groups. * *p* < 0.05. PwH: people with haemophilia; HA: haemophilic arthropathy; n.s.: not significant

The participants were instructed to wear a triaxial accelerometer – the wGT3X-BT (ActiGraph LLC, Pensacola, FL, USA) – on either side of their waist continuously for seven days except for during sleep and aquatic activities (Fig. 1a). They were explicitly advised to maintain their usual daily routines and normal levels of physical activity during the measurement period. The data were processed using ActiLife version 6.13.4 (ActiGraph LLC). The criterion for adopting data was that valid data were obtained for at least five days during the seven-day monitoring period. Although we encouraged continuous wear, the specific daily minimum wear time was not strictly defined as a prerequisite for inclusion. The epoch for count sampling was set to one minute.

On the basis of the criteria of Sasaki et al. [15], physical activity levels were quantified by calculating the proportion of total wear time spent in MVPA. Sedentary, light, and more intense activity levels were distinguished from MVPA. The wear time was calculated in minutes.

MVPA episodes lasting at least 10 consecutive minutes were defined as one bout (i.e., a single continuous session of at least moderate- intensity activity). This approach enabled the calculation of the ability to sustain at least 10 mins of MVPA. For participants who did not complete a bout of MVPA, the number of bouts was recorded as zero.

### Statistical analysis

The data were analysed descriptively by calculating the mean and standard deviation (SD) via statistical software. All the statistical analyses were performed using SPSS software (SPSS, Inc.; SPSS version 26.0 for Windows), and the threshold for statistical significance was set to 5%.

The normality of data distribution was assessed using the Shapiro-Wilk test, and the homogeneity of variance was evaluated using Levene’s test. When data did not meet the assumptions of normality or homogeneity of variance, the Kruskal–Wallis test was employed instead of one-way ANOVA. One-way ANOVA and the Kruskal‒Wallis test were used to compare parameters among the three groups (PwH with HA vs. PwH without HA vs. non-PwH). Then, all pairs of groups were contrasted within each factor to search for any significant heterogeneity detected by ANOVA. If a significant difference was found via one-way ANOVA, multiple comparisons were performed via Tukey’s test. If a significant difference was found via the Kruskal‒Wallis test, multiple comparisons were performed via the Steel–Dwass test.

## Results

### Background of PwH

Nineteen PwH with HA, 12 PwH without HA, and 15 non-PwH were enrolled. The severity of PwH, the AHS of the ankle and knee joints, and the methods of treatment are shown in Table 1. In the PwH with HA group, 15 participants had haemophilia A, and 4 participants had haemophilia B. In the PwH without HA group, 9 participants had haemophilia A, and 3 participants had haemophilia B. There were 16 severe cases, 1 moderate case, and 2 mild cases of haemophilia in the PwH with HA group and 8 severe cases, 2 moderate cases, and 2 mild cases of haemophilia in the PwH without HA group. In the PwH with HA group, 12 participants received prophylaxis via regular clotting factor agents (factor prophylaxis), 6 participants received prophylaxis via emicizumab (emicizumab prophylaxis), and one participant received on-demand treatment. In the PwH without HA group, 9 participants received factor prophylaxis, 2 participants received emicizumab prophylaxis, and 1 participant received on-demand treatment. Four participants in the PwH with HA group experienced joint pain. No participants in the PwH without HA group experienced joint pain. Regarding joint status, the 19 participants in the PwH with HA group exhibited established arthropathy (AHS stage 4 or 5) in the weight-bearing joints, as detailed in Table 1. None of the participants had a history of joint or intramuscular bleeding in the past year.

**Table 1:** Severity, treatment information, pain location, and Arnold–Hilgartner stages of the ankle and knee joints in PwH and non-PwH

							AHS			
							Ankle		Knee	
	ID	Age	Type	Severity	Method of treatment	Pain of lower limb	Left	Right	Left	Right
PwH with HA	1	51	A	Severe	Emicizumab prophylaxis	Right hip, Left knee	2	4	5	5
(*n* = 19)	2	74	A	Severe	Emicizumab prophylaxis	No pain	2	Arthrodesis	4	5
	3	57	A	Severe	Emicizumab prophylaxis	No pain	4	5	4	5
	4	52	A	Severe	Factor prophylaxis	Bilateral ankle	5	5	TKA	TKA
	5	60	A	Severe	Factor prophylaxis	Bilateral ankle	3	4	5	5
	6	64	B	Mild	On demand	No pain	2	4	2	3
	7	68	A	Moderate	Factor prophylaxis	No pain	0	4	4	4
	8	63	B	Severe	Factor prophylaxis	Left knee	5	5	2	2
	9	36	A	inhibitor	Emicizumab prophylaxis	No pain	5	5	3	5
	10	32	A	Severe	Emicizumab prophylaxis	No pain	2	4	2	0
	11	51	A	Severe	Factor prophylaxis	No pain	5	4	4	4
	12	27	A	Severe	Factor prophylaxis	No pain	4	2	1	1
	13	38	B	Severe	Factor prophylaxis	No pain	5	1	0	0
	14	28	A	Severe	Emicizumab prophylaxis	No pain	5	1	4	4
	15	36	A	Severe	Factor prophylaxis	No pain	4	Arthrodesis	1	1
	16	42	A	Severe	Factor prophylaxis	No pain	5	5	2	5
	17	44	A	Severe	Factor prophylaxis	Left knee, bilateral ankle	5	Arthrodesis	1	1
	18	68	B	Severe	Factor prophylaxis	No pain	5	5	2	TKA
	19	44	A	Mild	Factor prophylaxis	No pain	5	5	5	1
PwH without HA	20	53	A	Mild	Factor prophylaxis	No pain	0	0	1	1
(*n* = 12)	21	50	A	Moderate	On demand	No pain	0	0	0	0
	22	27	A	Severe	Emicizumab prophylaxis	No pain	0	0	0	0
	23	19	A	Moderate	Factor prophylaxis	No pain	0	0	0	0
	24	18	A	Severe	Factor prophylaxis	No pain	0	0	0	0
	25	21	A	Severe	Factor prophylaxis	No pain	0	0	0	0
	26	21	B	Severe	Factor prophylaxis	No pain	1	1	0	0
	27	22	B	Severe	Factor prophylaxis	No pain	1	1	0	0
	28	21	A	Severe	Emicizumab prophylaxis	No pain	0	0	0	0
	29	17	B	Mild	Factor prophylaxis	No pain	0	0	0	0
	30	23	A	Severe	Factor prophylaxis	No pain	1	1	1	1
	31	21	A	Severe	Factor prophylaxis	No pain	1	0	0	0
Non-PwH	32	28				No pain				
(*n* = 15)	33	38				No pain				
	34	20				No pain				
	35	26				No pain				
	36	30				No pain				
	37	30				No pain				
	38	43				No pain				
	39	63				No pain				
	40	28				No pain				
	41	58				No pain				
	42	53				No pain				
	43	28				No pain				
	44	26				No pain				
	45	34				No pain				
	46	28				No pain				

Abbreviations: AHS, Arnold-Hilgartner staging; HA, haemophilic arthropathy; PwH, people with haemophilia; TKA, total knee arthroplasty

### Differences in basic information and physical activity parameters among the three groups

The demographic and clinical characteristics of the participants, along with the results of the three-group comparison, are summarized in Table 2. While no significant difference was observed in BMI, there were significant differences among the three groups in age (*p* = 0.001), height (*p* = 0.001), and weight (*p* = 0.049). Specifically, the PwH without HA group was significantly younger and taller than the PwH with HA group. Additionally, the total wear time differed significantly among the groups (*p* = 0.013), with the PwH without HA group showing significantly shorter wear time than the other two groups, indicating lower adherence. These differences in baseline characteristics and wear time should be considered as potential confounders when interpreting physical activity data.

**Table 2:** Comparison of demographic characteristics and physical activity parameters among the three groups

	PwH groups			3-arm comparison		
	PwH with HA (*n* = 19)	PwH without HA (*n* = 12)	Non-PwH (*n* = 15)	*p* value	Statistic value	Comparion among groups
Age (years)	49.2 ± 14.4	26.1 ± 12.2	35.5 ± 12.9	0.001	11.454	†, †††
Height (cm)	165.5 ± 5.7	175.8 ± 7.2	170.4 ± 8.4	0.001	8.742	†
Weight (kg)	65.0 ± 8.8	73.3 ± 8.4	67.8 ± 12.0	0.049	3.398	
BMI (kg/m^2^)	23.7 ± 2.6	23.8 ± 2.9	23.4 ± 4.5	0.969	0.031	
**Physical activity parameters**						
Sedentary (%)	78.9 ± 12.5	78.8 ± 7.1	76.3 ± 5.8	0.549	0.612	
Light activity (%)	16.7 ± 9.6	14.5 ± 3.4	12.4 ± 3.2	0.114	2.351	
MVPA (%)	4.4 ± 5.2	6.7 ± 5.2	11.3 ± 3.4	0.001	11.117	††, †††
MVPA bouts (bout)	3.3 ± 5.7	4.5 ± 5.5	20.0 ± 10.2	0.001	16.148	††, †††
Wear time (minutes)	4,846.5 ± 1,899.8	3,152.2 ± 1,619.2	4,835.6 ± 956.2	0.013	5.230	†, ††

The data are expressed as the mean ± SD, with *p* < 0.05 indicating statistical significance

PwH: people with haemophilia, HA: haemophilic arthropathy, BMI: body mass index, MVPA: moderate to vigorous physical activity

† significant difference between PwH with HA and PwH without HA, *p* < 0.05

†† significant difference between the PwH without HA and the non-PwH group, *p* < 0.05

††† significant difference between the PwH with HA group and the non-PwH group, *p* < 0.05

**Fig. 2:**
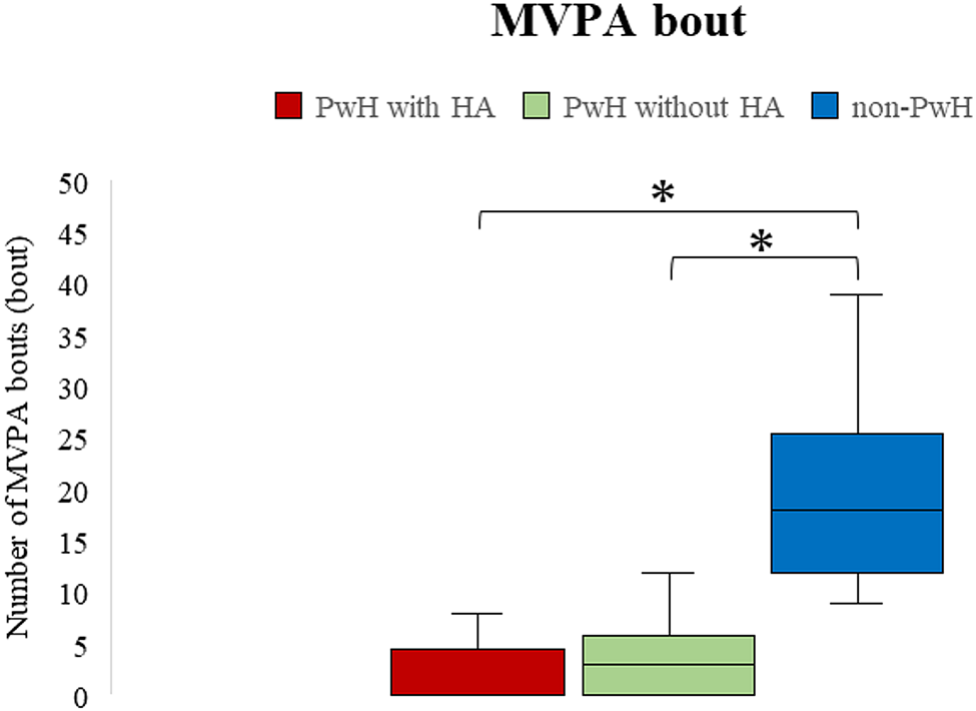
Number of MVPA bouts (≥ 10 mins) among the three groups. Box-and-whisker plots showing the distribution of MVPA bouts (continuous sessions of MVPA lasting ≥ 10 mins) across the PwH with HA (*n* = 19), PwH without HA (*n* = 12), and non-PwH (*n* = 15) groups. Compared with the PwH group, the non-PwH group recorded significantly more MVPA bouts. * *p* < 0.05. MVPA: moderate to vigorous physical activity; PwH: people with haemophilia; HA: haemophilic arthropathy

**Fig. 3:**
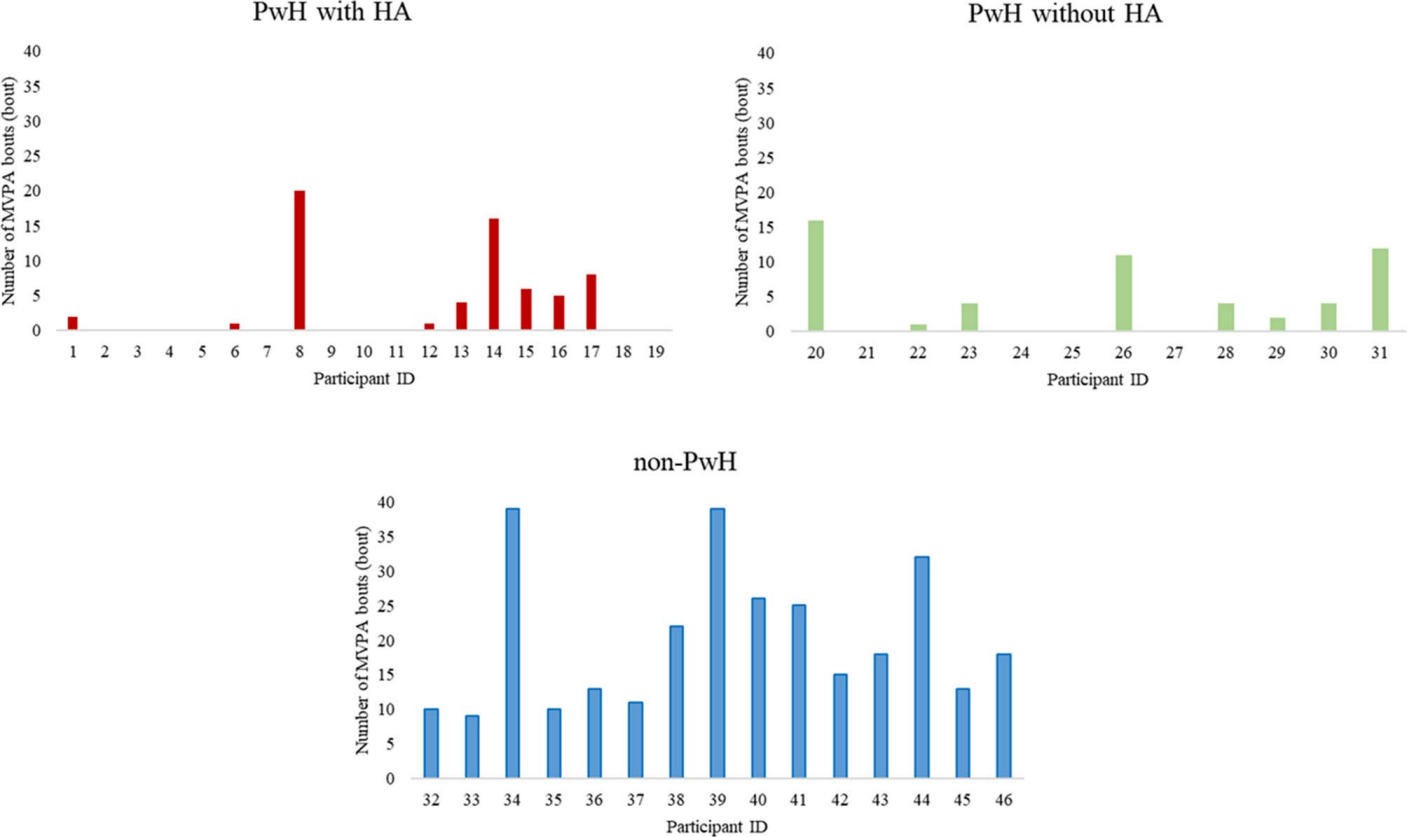
Individual MVPA bout frequency for each individual. Bar graph illustrating the number of MVPA bouts achieved by each individual across the three groups. PwH with HA (*n* = 19), PwH without HA (*n* = 12), and non-PwH (*n* = 15). Over half of the PwH with HA (10 of 19) and one quarter of the PwH without HA (3 of 12) recorded zero MVPA bouts. All non-PwH individuals achieved at least one MVPA bout. This figure highlights the large interindividual variability and the prevalence of physical inactivity among PwH, especially those with HA

Regarding physical activity parameters (Table 2; Fig. 1), no significant differences in the percentages of sedentary or light activity were observed across the three groups. However, the percentage of MVPA in the non-PwH group was significantly higher than in both the PwH with HA and PwH without HA groups (*p* < 0.001). Furthermore, the number of MVPA bouts was significantly higher in the non-PwH group compared to both PwH groups (Figs. 2 and 3). Notably, zero MVPA bouts were recorded by 52.6% (10/19) of participants in the PwH with HA group and 25% (3/12) in the PwH without HA group, whereas all participants in the non-PwH group recorded at least one MVPA bout.

## Discussion

This study is the first report from Japan to compare PA levels between PwH with HA and PwH without HA. The results demonstrated that PwH with HA group had significantly lower percentages of MVPA and lower frequencies of MVPA bouts than non-PwH group. Furthermore, PwH without HA group had significantly lower MVPA percentages and bout frequencies than non-PwH group, who were older than PwH without HA and thus were expected to have inherently lower PA levels [16]. These results suggest a general trend towards lower PA in PwH with and without HA. These findings align with previous international reports indicating that PwH, even when clinically stable—defined here as being free from acute bleeding episodes and maintained on a consistent treatment regimen without recent clinical deterioration—may restrict their PA owing to perceived bleeding risks or conditioned behavioural patterns [12].

Importantly, more than half of the PwH with HA reported zero bouts of MVPA during the weeklong monitoring, highlighting a critical inactivity issue in this population. Sustained MVPA is known to be associated with improved cardiovascular and musculoskeletal health, and its absence may place PwH with HA at greater risk of comorbidities, including sarcopenia and metabolic syndrome [17, 18]. Recent advances in haemophilia treatment have reduced symptomatic joint bleeding [7], but PA behaviour among PwH who have HA has not improved accordingly. It is noteworthy that the radiographic profiles of the two PwH groups were markedly distinct. In the PwH without HA group, most participants exhibited no joint abnormalities, with Arnold-Hilgartner scores of 0 or 1 in all assessed joints. Conversely, the majority of the PwH with HA group showed advanced joint deterioration (scores of 4 or 5) in multiple locations, often involving all four joints. Therefore, the differences in PA observed in this study reflect a comparison between individuals with nearly preserved joint integrity and those with extensive, multi-joint arthropathy, rather than a comparison between groups with only subtle differences in joint health.

Surprisingly, PwH without HA group had lower MVPA percentages than non-PwH group, despite being younger and having no structural joint damage. This finding suggests that factors unrelated to joint dysfunction, such as fear of bleeding imprinted from past bleeding experiences, low exercise self-efficacy, or avoidance of exercise due to explanations from physicians or caregivers about disease-causing bleeding, may contribute to activity limitations. In support of this, previous studies have reported that overprotective parenting and a lack of PA during childhood can have persistent effects on PA in adulthood [19, 20]. In our previous study, even in the absence of HA, PwH demonstrated gait abnormalities and reduced walking speed compared with the non-PwH group, which may also reflect reduced PA [13]. The significantly shorter wear time observed in the PwH without HA group is another important finding. While we did not collect participant-reported reasons for removing the device, this group was significantly younger than the other two groups and likely more engaged in varied social or occupational activities. The lower adherence in this younger population may reflect a busier lifestyle or perceived inconvenience of wearing the device during specific activities. Although a shorter wear time could potentially lead to an underestimation of total daily activity, the consistently lower MVPA and bout frequencies observed in this group—despite their youth—suggest that their reduced activity levels are a genuine finding rather than a mere reflection of shorter monitoring periods.

These findings underscore the importance of individualized, evidence-based PA promotion strategies for PwH, particularly those without HA. Given the documented safety and benefits of structured exercise in PwH [8], tailored interventions focusing on self-efficacy enhancement, education, and supervised physical training may be warranted. Furthermore, the use of objective tools such as accelerometry and wearable impact sensors enables clinicians to monitor activity patterns and the symmetry of limb loading in real-life settings, which can guide rehabilitation and preventative strategies aimed at preserving musculoskeletal health in modern PwH.

This study has several limitations. First, the small sample size, particularly in PwH without HA, may limit generalizability and statistical power to detect subtle group differences. Second, factors related to PA measurement may have influenced the results: the lack of a strict daily minimum wear time, potential imbalances between weekdays and weekends, and the difficulty in distinguishing non-wear time from sedentary behavior. Crucially, as accelerometers cannot be worn during aquatic activities, any MVPA performed in the water (e.g., swimming) was not captured. Given that swimming is frequently recommended for PwH to reduce joint stress, this may have led to an underestimation of their actual PA levels. Third, although we used a standardized algorithm to determine wear time, it remains challenging to perfectly distinguish prolonged sedentary behavior from periods when the tracking device was not worn. This potential misclassification of non-wear time as sedentary behavior should be considered when interpreting the results. Fourth, reactivity bias from using activity trackers might have inflated PA levels, particularly in healthy controls, potentially accentuating differences between groups. Fifth, while participants were recruited year-round, the potential influence of seasonality and extreme weather on activity patterns was not fully accounted for. **Sixth**, the healthy control group consisted of employees from a single pharmaceutical company, which may not fully represent the general population. Although we applied the same exclusion criteria—including the exclusion of high-activity occupations—to ensure comparability with the PwH group, the socioeconomic background or health literacy of this specific cohort might have influenced the observed physical activity levels. Seventh, the Arnold-Hilgartner system may underestimate early-stage arthropathy undetectable by X-ray; thus, the “without HA” group might include participants with subclinical changes only identifiable via MRI or ultrasound. Eighth, the lack of upper-limb assessment (e.g., HJHS) is a limitation, as elbow arthropathy can also restrict overall activity. Ninth, the absence of a strict daily wear time threshold (e.g., 10 h) may affect comparability with other studies and should be noted when evaluating the results.

## Conclusions

Our findings revealed that compared with healthy participants, haemophilia patients – regardless of the presence of arthropathy – exhibit significantly reduced PA. These findings suggest a general reduction in PA levels among PwH regardless of joint damage, which is likely influenced by psychological and behavioural factors. Given that more than half of PwH with HA reported zero bouts of sustained MVPA despite advances in prophylactic treatments, there is a critical need for individualized, evidence-based PA interventions. Tailored strategies focusing on education, self-efficacy, and objective monitoring may help PwH meet the physical activity levels recommended by international guidelines, thereby contributing to their long-term musculoskeletal health.

## Data Availability

The data that support the findings of this study are available from the corresponding author upon reasonable request.

## Abbreviations

AHS: Arnold–Hilgartner staging
BMI: Body mass index
HA: Haemophilic arthropathy
MVPA: Moderate-to-vigorous physical activity
PA: Physical activity
PwH: People with haemophilia
QOL: Quality of life
SD: Standard deviation

## References

[1] Srivastava A, Santagostino E, Dougall A, Kitchen S, Sutherland M, Pipe SW, et al. WFH guidelines for the management of hemophilia, 3rd edition. Haemophilia. 2020;26:1–158. 10.1111/hae.14046 32744769

[2] Knobe K, Berntorp E. Haemophilia and joint disease: pathophysiology, evaluation, and management. J Comorb. 2011;1:51–9. 29090136 10.15256/joc.2011.1.2 PMC5556421

[3] Riley RR, Witkop M, Hellman E, Akins S. Assessment and management of pain in haemophilia patients. Haemophilia. 2011;17:839–45. 21645179 10.1111/j.1365-2516.2011.02567.x

[4] Witkop M, Neff A, Buckner TW, Wang M, Batt K, Kessler CM, et al. Self-reported prevalence, description and management of pain in adults with haemophilia: methods, demographics and results from the pain, functional impairment, and quality of life (P-FiQ) study. Haemophilia. 2017;23:556–65. 28419637 10.1111/hae.13214

[5] Stromer W, Pabinger I, Ay C, Crevenna R, Donnerer J, Feistritzer C, et al. Pain management in hemophilia: expert recommendations. Wien Klin Wochenschr. 2021;133:1042–56. 33661391 10.1007/s00508-020-01798-4 PMC8500904

[6] Manco-Johnson MJ, Soucie JM, Gill JC. Prophylaxis usage, bleeding rates, and joint outcomes of hemophilia, 1999 to 2010: a surveillance project. Blood. 2017;129:2368–74. 28183693 10.1182/blood-2016-02-683169 PMC5409445

[7] Oldenburg J, Mahlangu JN, Kim B, Schmitt C, Callaghan MU, Young G, et al. Emicizumab prophylaxis in hemophilia a with inhibitors. N Engl J Med. 2017;377:809–18. 28691557 10.1056/NEJMoa1703068

[8] Strike K, Mulder K, Michael R. Exercise for haemophilia. Cochrane Database Syst Rev. 2016;12:CD011180. 27992070 10.1002/14651858.CD011180.pub2 PMC6463808

[9] Calatayud J, Pérez-Alenda S, Carrasco JJ, Cruz-Montecinos C, Andersen LL, Bonanad S, et al. Safety and Effectiveness of Progressive Moderate-to-Vigorous Intensity Elastic Resistance Training on Physical Function and Pain in People With Hemophilia. Phys Ther. 2020;100:1985–96. 10.1093/ptj/pzaa106 32525975

[10] World Health Organization. WHO guidelines on physical activity and sedentary behaviour. Geneva: World Health Organization; 2020. 33369898

[11] Negrier C, Seuser A, Forsyth A, Lobet S, Llinas A, Rosas M, et al. The benefits of exercise for patients with haemophilia and recommendations for safe and effective physical activity. Haemophilia. 2013;19:487–98. 23534844 10.1111/hae.12118

[12] Kennedy M, O’Gorman P, Monaghan A, Lavin M, O’Mahony B, O’Connell NM, et al. A systematic review of physical activity in people with haemophilia and its relationship with bleeding phenotype and treatment regimen. Haemophilia. 2021;27:544–62. 33751742 10.1111/hae.14282 PMC8359343

[13] Mawarikado Y, Sakata A, Inagaki Y, Harada S, Tatsumi K, Matsumoto N, et al. Force-sensing treadmill gait analysis system can detect gait abnormalities in haemophilia patients without arthropathy. Haemophilia. 2024;30:780–90. 38507270 10.1111/hae.14987

[14] Arnold WD, Hilgartner MW. Hemophilic arthropathy. Current concepts of pathogenesis and management. J Bone Joint Surg Am. 1977;59:287–305. 849938

[15] Sasaki JE, John D, Freedson PS. Validation and comparison of ActiGraph activity monitors. J Sci Med Sport. 2011;14:411–6. 21616714 10.1016/j.jsams.2011.04.003

[16] Haskell WL, Lee IM, Pate RR, Powell KE, Blair SN, Franklin BA, et al. Physical activity and public health: updated recommendation for adults from the American College of Sports Medicine and the American Heart Association. Circulation. 2007;116:1081–93. 17671237 10.1161/CIRCULATIONAHA.107.185649

[17] Piercy KL, Troiano RP, Ballard RM, Carlson SA, Fulton JE, Galuska DA, et al. The physical activity guidelines for Americans. JAMA. 2018;320:2020–8. 30418471 10.1001/jama.2018.14854 PMC9582631

[18] Cruz-Jentoft AJ, Bahat G, Bauer J, Boirie Y, Bruyère O, Cederholm T, et al. Sarcopenia: revised European consensus on definition and diagnosis. Age Ageing. 2019;48:601. 31081853 10.1093/ageing/afz046 PMC6593317

[19] Bérubé S, Ogez D, Aramideh J, Amesse C, Bourque CJ, Longpré C, et al. It’s difficult to say no: development of a parenting booklet about physical activity restrictions and recommendations in pediatric hemophilia. Health Psychol Open. 2021;8:20551029211039920. 34881045 10.1177/20551029211039920 PMC8647236

[20] Goto M, Takedani H, Yokota K, Haga N. Strategies to encourage physical activity in patients with hemophilia to improve quality of life. J Blood Med. 2016;7:85–98. 27274330 10.2147/JBM.S84848 PMC4876843

